# Effect of combining low intensity-extracorporeal shockwave therapy with platelet-rich plasma among female patients with interstitial cystitis: a prospective single-arm pilot study

**DOI:** 10.3389/fbioe.2026.1801578

**Published:** 2026-05-28

**Authors:** Cheng-Yu Long, Chieh-Yu Chang, I-Chieh Sung, Liang-Ying Ke, Hui-Yu Chuang

**Affiliations:** 1 Department of Obstetrics and Gynecology, Kaohsiung Medical University Hospital, Kaohsiung Medical University, Kaohsiung, Taiwan; 2 Department of Obstetrics and Gynecology, Kaohsiung Municipal Ta-Tung Hospital, Kaohsiung, Taiwan

**Keywords:** bladder pain syndrome, interstitial cystitis, low-intensity extra-corporeal shockwave therapy, platelet-rich plasma, tissue regeneration

## Abstract

**Background:**

Interstitial cystitis (IC) is a disease comprised of multiple symptoms, included persistent bladder pain syndrome (BPS), frequent urination, and urgency without getting infection or other identifiable causes.

**Methods:**

In our study, 25 female patients diagnosed with IC/BPS, the patients were performed low-intensity extracorporeal shockwave therapy (LI-ESWT) once a week for 8 weeks. The application sites for LI-ESWT are located above the pubic bone, in the middle and on both sides, with each session lasting about 20–30 min. High dosage of platelet-rich plasma (PRP) injections were administered in weeks 1, 5, and 9. All subjects had a baseline, 1-month and 6-month post-treatment assessment that included validated questionnaires and voiding diaries. Urodynamic studies were conducted at baseline and the 6-month follow-up after treatment.

**Results:**

We observed an 80% efficacy rate. Then, we showed significant improvements in VAS, OABSS, UDI-6, IIQ-7, and POP at the 1-month follow-up. However, only the ICIQ showed significant improvement at the 6-month follow-up. We further examined the urodynamic parameters and MCC revealed significant difference at 6-month follow-up. The voiding diaries, which tracks the number of urinations, voided volume, maximum volume, and nocturia significant improved at 1-month follow-up while only maximum volume showed improvement at the 6-month follow-up. Finally, ICSI, ICPI, and OSS, all showed significant differences at both the 1-month and 6-month follow-ups.

**Conclusion:**

The results of our study suggested that the combination treatment of PRP and LI-ESWT might show a therapeutic efficacy to IC/BPS patients, However, the follow-ups at 1 month and 6 months post-treatment suggested that the treatment needs to be administered again over time to maintain its effectiveness.

## 1 Introduction

Interstitial cystitis (IC) is a disease comprising multiple symptoms, including persistent bladder pain syndrome (BPS), frequent urination, and urgency without infection or other identifiable causes. The global prevalence of IC/BPS varies significantly, ranging from 2.7% to 6.5% in women based on Asian studies and up to 4.2% in European populations, reflecting regional differences in diagnostic criteria and awareness levels. In the United States, the prevalence is estimated at 1.08% (95% CI: 0.03, 2.13). In Asia, prevalence appears to be lower, with reports from Japan indicating a rate of 1.0% and studies from Korea estimating 0.26% (Lee et al., 2021). A Taiwan National Database analysis showed an increasing trend, from 21.8 per 100,000 in 2002 to 40.2 per 100,000 in 2013, suggesting greater recognition and diagnosis over time (Chung et al., 2013). This broader perspective highlights the global burden of IC/BPS, underscoring the need for effective treatments (Anger et al., 2022). To deal with this disease, there is no single treatment that works for every patient. Current treatment options are divided into conservative treatment including stress management, change in daily habit, pelvic floor muscle exercise or physical therapy (Clemens et al., 2022), oral medication such as histamine inhibitors, pentosan polysulfate sodium or tricyclic antidepressants (Yu and Kuo, 2024; Arıkan and Çakıroğlu, 2023; Shan et al., 2019), intravesical physical treatment or surgery; nevertheless, there are some problems in the current treatments such as the inconsistency of therapeutic effect and adverse effects from oral medication, short duration of the treatment effect, risk of surgery, etc., (Chen et al., 2021; Garzon et al., 2020). Accordingly, other treatment options have been developed such as laser therapy, platelet-rich plasma (PRP) or Low-intensity extracorporeal shock wave therapy (LI-ESWT) (Okui et al., 2020; Kuo, 2023; Chuang et al., 2020).

One previous study used PRP as a novel therapy for IC/BPS. Lee et al. (2022) showed that monthly intravesical PRP injections had therapeutic effects in treating IC/BPS where significant beneficial effects in clinical symptoms and urothelial ultra-structural defects were found, particularly in tight junctions (Lee et al., 2022). Additionally, Jiang et al. (2022) demonstrated that high-dose PRP injected over 20 or 40 sites significantly ameliorated IC symptoms, VAS score and flow rate at 3 months after single high-dose injection. In contrast, there were no significant differences in other groups in ICSI, ICPI and GRA at 6 months (Jiang et al., 2022), while apart from PRP, LI-ESWT was also used in treating IC/BPS.

Some studies have also investigated the therapeutic effects of LI-ESWT in IC/BPS. Jhang L. S. et al. (2023) demonstrated that 8 weeks of LI-ESWT treatment could significantly alleviated ICSI, voiding diary and urodynamics parameter at 1-month or 1-year follow-up assessments, but no effect was shown in all urodynamic parameters (Jhang L. S. et al., 2023). The main reason they used LI-ESWT in IC/BPS was due to its anti-inflammatory and anti-apoptotic effects. Shen et al. (2021) showed that LI-ESWT could significantly reduce VAS and OSS score compared to placebo group at 4–12 weeks; however, some urinary markers including VEGF and IL-9 were significantly changed at 4 weeks and showed no persistent effect at 12 weeks (Shen et al., 2021). Chuang et al. (2020) also demonstrated that more patients in LI-ESWT group improved in the VAS ≥ 3 than placebo at 4 or 12 weeks after post-treatment, with the frequency improving more in the LI-ESWT group (Chuang et al., 2020). To sum up the previous reports, although some improvement effects were seen in PRP or LI-ESWT treatments, no study investigated the combined therapy of PRP plus LI-ESWT in IC/BPS.

LI-ESWT and PRP are emerging regenerative therapies that have been explored in various fields, including urology, orthopedics, and dermatology, owing to their ability to promote healing and reduce inflammation via their therapeutic effects being mediated by several biological mechanisms. LI-ESWT exerts its effects by inducing mechanotransduction, which activates cellular pathways leading to angiogenesis, tissue re-modeling, and inflammation modulation (Liu et al., 2019), while PRP therapy provides a concentrated source of platelet-derived growth factors that facilitate wound healing, collagen synthesis, and cell proliferation. Key bioactive molecules in PRP, including plate-let-derived growth factor (PDGF), transforming growth factor-beta (TGF-β) and insulin-like growth factor (IGF-1), promote angiogenesis, extracellular matrix remodeling, and urothelial repair (Lubkowska et al., 2012).

The rationale of combining these two methods is that PRP could provide the necessary bioactive molecules to promote tissue repair while we believe LI-ESWT could enhance this process through mechanical stimulation, thereby facilitating the absorption of PRP and expanding its effective area. Thus, our study intended to combine these two therapies to produce a synergistic effect in accelerating tissue repair, reducing inflammation and relieving pain in IC/BPS. To our best knowledge, our study is the first to apply PRP plus LI-EWST on IC/BPS and validate its therapeutic effect on urinary symptoms.

## 2 Materials and methods

### 2.1 Subject basic characteristics

**FIGURE 1:**
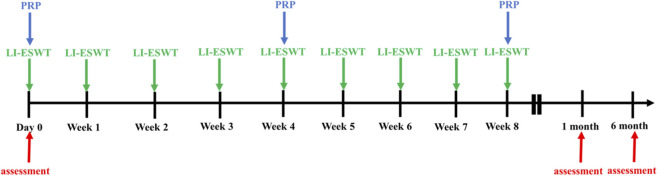
The treatment protocol of PRP plus LI-ESWT in our study. The treatment protocol in our study was shown. The patients underwent LI-ESWT once a week for 8 weeks. LI-ESWT was applied above the pubic bone, centrally and on both sides, with each session lasting about 20–30 min. High doses of PRP injections were administered in weeks 1, 5, and 9.

Our current study recruited twenty-five female patients diagnosed with IC/BPS who underwent low-intensity extracorporeal shockwave therapy (LI-ESWT) from March 2020 to March 2023. The diagnostic criteria for IC/BPS were based on the ESSIC guidelines and the exclusion of similar diseases (Hanno and Sant, 2001; van de Merwe et al., 2008). Our study was approved by the Institutional Review Board and Ethics Committee of the Faculty of Kaohsiung Medical University Hospital (KMUHIRB-F(I)-20200050). Patients were consecutively enrolled from our department based on eligibility criteria. This study was designed as a prospective, single-arm exploratory pilot study to evaluate the feasibility, safety, and preliminary efficacy of combined PRP and LI-ESWT in patients with IC/BPS. Given the absence of prior studies investigating this combination therapy, a non-randomized design without a control group was considered appropriate as an initial proof-of-concept. All patients provided written informed consent before participation. Of the 25 recruited patients, all completed the initial treatment phase and follow-up assessments. No patients were lost to follow-up, ensuring a complete dataset for statistical analysis. The patients who participated in our study were assessed using the O’Leary–Saint symptom score (OSS), which comprised the IC symptom index (ICSI), IC problem index (ICPI), visual analog scale (VAS) for bladder pain and voiding diary were acquired at the beginning of the study, 1 month after treatment, and 6 months after treatment. The IC/BPS patients were examined routine urine tests, urodynamic studies, and questionnaires, including the overactive bladder symptom score (OABSS) and urinary distress inventory-6 (UDI-6), incontinence impact questionnaire (IIQ-7), international consultation of incontinence questionnaire (ICIQ-SF), pelvic organ prolapse distress inventory (POPDI-6). The treatment protocol in our study is shown in Figure 1. In brief, the patients were performed LI-ESWT (STORZ MEDICAL EvoTronTM, GA, 3,000 pulse, 0.25 mJ/mm2) once a week for 8 weeks. The application sites for LI-ESWT are located above the pubic bone, in the middle and on both sides, with each session lasting about 20–30 min. High doses of PRP injections were administered in weeks 1, 5, and 9.

### 2.2 Preparation of platelet rich plasma

The preparation was based on our previous study (Long et al., 2021). In brief, we drew 10 mL of the patient’s blood and injected it into an Aeon Acti-PRP tube. After centrifugation, the tube was gently shaken to mix the plasma layer and an 18G needle was used to extract high-concentration (1.6X) platelet plasma under laminar flow conditions that was then mixed with 20 mL of saline solution. After disinfecting the vulva and urethral opening, the physician inserted a No. 10 urinary catheter into the urethra to drain residual urine. The prepared PRP and saline mixture was then injected into the bladder.

### 2.3 Assessment of female urinary symptoms in subjects

The assessment of female lower urinary tract symptoms included the OABSS, UDI-6, IIQ-7, ICIQ, and POPDI were acquired at the beginning of the study, 1- and 6-months post treatment. OABSS, as described by Homma et al. (2006), included four questions related to OAB, with a maximum score range of 2–5. The total score ranged from 0 to 15 points, with higher scores indicating more severe symptoms. UDI-6 and IIQ-7 were referenced from previous literature (Shumaker et al., 1994; Corcos et al., 2002). The ICIQ contained four questions with a maximum score range of 5–10, and the total score was 21. POPDI, as described by Barber et al. (2005), consisted of six questions rated on a scale of 0–3. The Global Response Assessment (GRA) scale was used to evaluate patient-perceived therapeutic success following PRP + LI-ESWT treatment. The GRA is a validated, patient-reported scale commonly used in clinical studies on IC/BPS to measure subjective improvement as in a previous study (Mishra, 2022). Patients were asked to rate their overall symptom change compared to baseline using a 7-point Likert scale: +3: Markedly improved; +2: Moderately improved; +1: Slightly improved; 0: No change; −1: Slightly worse; −2: Moderately worse; −3: Markedly worse; for this study, therapeutic success was defined as a GRA score of +2 or higher (moderately or markedly improved) at follow-up assessments (1, 3, and 6 months after treatment). This threshold was chosen based on prior IC/BPS studies, where GRA ≥ +2 correlated with meaningful clinical improvement in pain and urinary symptoms (Wang et al., 2019).

### 2.4 Urodynamics study of patients

Urodynamic studies, including non-instrumented uroflowmetry, filling and voiding cystometry, and urethral pressure profilometry, were performed according to the recommendations by the International Continence Society (Reynard et al., 1998) with a 6-channel urodynamic monitor (MMS; UD2000, Enschede, Netherlands). Uninhibited detrusor contractions during filling cystometry were considered positive for detrusor overactivity (DO).

### 2.5 Statistical analysis

Data were represented as mean ± standard deviations with a p < 0.05 indicating a statistically significant difference. Statistical analyses were performed using IBM SPSS Statistical Software version 20.0 ed. The normality of data distribution was assessed using the Shapiro-Wilk test, given the small sample size. If normality was satisfied, paired t-tests were performed for two related units on a continuous outcome. For handling missing data, a last observation carried forward (LOCF) approach was used when follow-up data were unavailable for specific time points, as this method minimizes bias in small-sample studies. A p-value of less than 0.05 indicated statistical significance.

## 3 Results

### 3.1 Patient’s basic characteristics

We recruited a total of 25 patients in the prospective study. All enrolled patients had previously received at least one form of conservative or medical therapy for IC/BPS (e.g., oral medication or intravesical therapy), but had either inadequate response or intolerable side effects, thus qualifying them for inclusion in this prospective study. Their essential characteristics are listed in Table 1 as having a mean age of 45.1 ± 10.8 with aver-age body mass index (BMI) of 22.0 ± 3.2, while two (8%) of them were in menopause and one (4%) was undergoing hormone therapy during the trial. Besides, one (4%) of them had diabetes mellitus. Among these, the history of all IC patients was 6.1 ± 5.6 years. We identified global response assessment (GRA) 2, 3 as a successful treatment rate with twenty people (80%) evidenced in the study. No severe side effects were observed in this study.

**TABLE 1:** Clinical background of women with interstitial cystitis.

Parameter (n = 25)	Value
Mean age (years)	45.1 ± 10.8
Mean parity	1.3 ± 1.1
Mean BMI (kg/m^2^)	22.0 ± 3.2
Menopause	2 (8)
Current hormone therapy	1 (4)
Diabetes mellitus	1 (4)
Hypertension	1 (4)
History of hysterectomy	0
History of IC (years)	6.1 ± 5.6
Follow-up (months)	1, 6
Success rate (GRA 2,3)	20 (80%)

Data are given as mean ± standard deviation, or n (%).

### 3.2 The effect of PRP plus LI-ESWT on urinary symptoms and quality of life questionnaire in IC patients

We intended to evaluate the therapeutic effect of platelet-rich plasma (PRP) plus Low-intensity extracorporeal shock wave therapy (LI-ESWT) on urinary symptoms in IC patients. The patients were treated with PRP once a week for 8 weeks and received LI-ESWT once per month three times. The VAS score, OABSS, UDI-6, IIQ-7, ICIQ, and POP were all acquired at the beginning, 1 month, and 6 months after the last treatment and are shown in Table 2. In the 1-month follow-up, VAS score was significantly improved from 5.4 ± 2.3 to 1.6 ± 1.7 (p = 0.004); OABSS was decreased considerably from 6.1 ± 3.6 to 3.9 ± 2.8 (p = 0.05); UDI-6 was significantly reduced from 42.9 ± 17.0 to 29.4 ± 11.0 (p = 0.018); POP significantly improved from 11.4 ± 5.0 to 5.6 ± 3.5 (p = 0.036); while ICIQ slightly changed from 3.3 ± 3.8 to 1.7 ± 2.2. However, the VAS, OABSS, UDI-6, IIQ-7, and POP scores slightly regressed back to the original baseline level. Only ICIQ significantly changed from 3.3 ± 3.8 to 2.5 ± 2.6 (p = 0.046) (Table 2).

**TABLE 2:** Effect of PRP plus LI-ESWT on urinary symptoms related questionnaires at 1-month and 6-months follow-ups.

n = 25	Pre	Post-treatment 1m	p value	Post-treatment 6m	p value
VAS	5.4 ± 2.3	1.6 ± 1.7	0.004*	3.3 ± 2.4	0.5
OABSS	6.1 ± 3.6	3.9 ± 2.8	0.05*	3.8 ± 1.6	0.343
UDI-6	42.9 ± 17.0	29.4 ± 11.0	0.018*	30.6 ± 6.2	0.195
IIQ-7	79.6 ± 15.2	47.6 ± 32.5	0.013*	57.1 ± 49.1	0.47
ICIQ	3.3 ± 3.8	1.7 ± 2.2	0.111	2.5 ± 2.6	0.046*
POP	11.4 ± 5.0	5.6 ± 3.5	0.036*	5.0 ± 3.2	0.207

VAS: visual analogue scale; OABSS: overactive bladder symptom score; UDI-6: urinary distress inventory-6; IIQ-7: incontinence impact questionnaire; ICIQ: international consultation on incontinence questionnaire; POP: pelvic organ prolapse quantification system.

Values are given as mean ± standard deviation.

* Statistical significance; paired t-test. *, *p* < 0.05 compared to Pre-treatment value.

### 3.3 The effect of PRP plus LI-ESWT on urodynamic studies in IC patients

We then examined the urodynamics parameter on IC patients in a 6-month follow-up. Among all parameters (Qmax: maximum flow rate; RU: residual urine; Vfst: bladder volume at first sensation to void; MCC: maximum cystometric capacity; Pdet (cm H2O): maximal detrusor pressure at Qmax; MUCP: maximum urethral closure pressure; FUL: functional urethral length; UCA: urethral closure pressure), only MCC significantly increased at the 6-month follow-up from 261.4 ± 75.9 to 341.6 ± 38.0 after the PRP plus LI-ESWT treatment, while other parameters did not show significant changes (Table 3).

**TABLE 3:** Effect of PRP plus LI-ESWT on urodynamic studies at 6-month follow-up.

n = 25	Pre	Post-treatment 6m	p-value
Qmax (mL/s)	14.5 ± 3	17.7 ± 5.9	0.17
RU (mL)	40.4 ± 35.2	30.2 ± 37.8	0.33
Vfst (mL)	78.2 ± 53.6	89.6 ± 36.8	0.06
MCC (mL)	261.4 ± 75.9	341.6 ± 38.0	0.045*
Pdet (cm H_2_O)	30.4 ± 15.7	18.2 ± 16.4	0.28
MUCP (cm H_2_O)	86.2 ± 20.3	65.6 ± 24.5	0.07
FUL (mm)	24.4 ± 2.9	24.2 ± 3.2	0.75
UCA (mm. cm H_2_O)	1090.2 ± 229.5	1051.5 ± 135.2	0.37

Qmax: maximum flow rate; RU: residual urine; Vfst: = bladder volume at first sensation to void; MCC: maximum cystometric capacity; Pdet (cm H_2_O): maximal detrusor pressure at Qmax; MUCP: maximum urethral closure pressure; FUL: functional urethral length; UCA: urethral closure pressure.

Values are expressed as mean ± standard deviation.

* Statistical significance; paired t-test. *, *p* < 0.05 compared to Pre-treatment value.

### 3.4 The effect of PRP plus LI-ESWT on voided diaries in IC patients

We then acquired voided diaries from patients. Numbers of urination, voided volume, maximum volume, and nocturia were recorded, with results demonstrating that PRP plus LI-ESWT treatment significantly reduced the numbers of urination from 16.5 ± 6.5 to 14.2 ± 6.6 (p = 0.015) at 1-month follow-up, although this slightly regressed to 15.0 ± 9.0 (p = 0.08). Besides, the voided volume significantly increased from 116.4 ± 64.4 to 144.6 ± 75.3 (p < 0.001) at 1-month follow-up, but it also regressed from 144.6 ± 75.3 to 122.9 ± 75.3 at the 6-month follow-up. The maximum volume was likewise observed, with data showing that the treatment of PRP plus LI-ESWT significantly upregulated the maximum volume of urine from 225.4 ± 95.8 to 270.8 ± 109.9 (p = 0.01) at the 1-month follow-up with a significant increase also recorded at the 6-month follow-up (225.4 ± 95.8 to 253.8 ± 103.6) (p = 0.022). Finally, nocturia did not show a significant change at both 1-month (2.0 ± 1.1) and 6-month (2.2 ± 1.7) follow-up assessments (Table 4).

**TABLE 4:** Voiding diaries at 1-month and 6-month follow-ups.

n = 25	Pre	Post-treatment 1m	p-value	Post-treatment 6m	p-value
Numbers of urination	16.5 ± 6.5	14.2 ± 6.6	0.015*	15.0 ± 9.0	0.08
Voided volume (mL)	116.4 ± 64.4	144.6 ± 75.3	<0.001*	122.9 ± 78.5	0.69
Maximum volume (mL)	225.4 ± 95.8	270.8 ± 109.9	0.01*	253.8 ± 103.6	0.022*
Nocturia	2.4 ± 1.7	2.0 ± 1.1	0.11	2.2 ± 1.7	0.57

Data are given as mean ± standard deviation, or n (%).

* Statistical significance; paired t-test. *, *p* < 0.05 compared to Pre-treatment value.

### 3.5 The effect of PRP plus LI-ESWT on IC related questionnaires at pretreatment, 1 month after treatment and 6 months after treatment

Some IC-related questionnaires, including ICSI, ICPI, and OSS, were acquired at the beginning of the study, 1 month after treatment, and 6 months after treatment. Our results showed that PRP plus LI-ESWT treatment significantly improved ICSI from 12.0 ± 3.7 to 6.1 ± 5.1 at the 1-month follow-up (p = 0.02); moreover, the decrease in ICSI score was also observed in the 6-month follow-up from 12.0 ± 3.7 to 4.9 ± 3.8 (p = 0.03). The ICPI data also showed that the co-treatment of PRP plus LI-ESWT significantly reduced from 10.8 ± 4.1 to 3.6 ± 3.1 (p = 0.012) at the 1-month follow-up and 10.8 ± 4.1 to 3.8 ± 1.5 (p = 0.024) at the 6-month follow-up respectively, while OSS also showed the same trend as ICSI and ICPI. The co-treatment of PRP plus LI-ESWT significantly attenuated OSS from 24.8 ± 7.7 to 10.1 ± 6.5 (p = 0.01) at the 1-month follow-up (p = 0.01) and 24.8 ± 7.7 to 8.7 ± 4.3 (p = 0.015) at the 6-month follow-up (Table 5).

**TABLE 5:** Questionnaires of IC symptoms (ICSI, ICPI, OSS) for the pre-trial patients, 1-month follow-up and 6-month follow-up (interstitial cystitis symptom index, ICSI; interstitial cystitis problem index, ICPI; O’Leary-saint symptom score, OSS).

n = 25	Pre	Post-treatment 1m	p-value	Post-treatment 6m	p-value
ICSI	12.0 ± 3.7	6.1 ± 5.1	0.02*	4.9 ± 3.8	0.03*
ICPI	10.8 ± 4.1	3.6 ± 3.1	0.012*	3.8 ± 1.5	0.024*
OSS	24.8 ± 7.7	10.1 ± 6.5	0.01*	8.7 ± 4.3	0.015*

ICSI: interstitial cystitis symptom index; ICPI: interstitial cystitis problem index; OSS: O’Leary-sant symptom score.

Data are given as mean ± standard deviation.

* Statistical significance; paired t-test. *, *p* < 0.05 compared to Pre-treatment value.

## 4 Discussion

To provide a global perspective, we included prevalence data from multiple regions, underscoring the worldwide burden of IC/BPS. This broader context highlights the potential global impact of our findings, especially given the significant short-term efficacy demonstrated in our study (80% success rate at 6 months). By combining PRP and LI-ESWT, our approach not only provides symptomatic relief but also addresses the underlying inflammatory and regenerative processes in IC/BPS. Our study firstly investigated the therapeutic effects of PRP plus LI-ESWT treatment in IC/BPS patients, with the data showing that the ratio of GRA ≥ 2 among all patients was 80%; moreover, the PRP plus LI-ESWT treatment significantly improved VAS, OABSS, UDI-6, IIQ-7 and POP questionnaires outcomes in IC/BPS patients during the 1-month follow-up. Furthermore, the urodynamics studies also depicted that the combined treatment significantly in-creased MCC, while the data of voiding diaries demonstrated that PRP plus LI-ESWT treatment ameliorated the number of urinations, voided volume, maximum volume at 6-month follow-up and maximum volume at 6-month follow-up. Finally, the combined treatment showed a significant improvement in ICSI, ICPI and OSS at both 1-month and 6-months follow-ups. Besides our study, other previous studies have also discussed the feasibility of combining PRP and LI-ESWT (Alkhabbaz et al., 2024; Seabaugh et al., 2017; Kuo et al., 2024; Chang et al., 2020).

First, an *in vitro* study investigated the effect of shock wave on PRP. Seabaugh et al. (2017) showed that the treatment of ESWT-standard probe and ESWT-power probe could significantly increase the concentration of PRP in TGF-β1 and PDGF-BB20, while Chang et al. (2020) demonstrated the effect of PRP plus LI-ESWT on moderate carpal tunnel syndrome (CTS). Their results depicted that combined therapy showed beneficial effects on the Boston CTS questionnaire at 1-month follow-up and distal motor latency at 3-month follow-up compared to the control group, although the combination of PRP with one-session ESWT was not superior to PRP alone in treating moderate CTS (Chang et al., 2020). Moreover, Kuo et al. (2024) demonstrated that not only PRP alone but also PRP combined with ESWT showed significant improvements in VAS, Constant–Murley score, degrees of forward flexion, abduction, internal rotation, external rotation and range of shoulder motion at 6-month follow-up, while this combination also showed enhancement in forward flexion and abduction at 1-month follow-up. Additionally, a significant increase was found within the run-of-bear movement at 6-month follow-up. In serum analysis, the concentrations of inflammation modulator, S100A8 and S100A9, were both lower in the ESWT + PRP group (Kuo et al., 2024).

Our data depicted that PRP plus LI-ESWT revealed a significant short-term efficacy (1 month), although the effectiveness began to diminish gradually after 6 months. To address this issue, increasing the number of sessions or combining with other therapies could be considered to prolong the duration of its efficacy. From a biological perspective, the combination of PRP and LI-ESWT has multiple potential mechanisms of action. PRP promotes tissue repair and has anti-inflammatory effects (Abdul Ameer et al., 2018; Alsousou et al., 2013), while LI-ESWT enhances blood flow and tissue regeneration17,18 consequently, the synergistic effects of these two therapies could therefore provide better symptoms improvement and long-term efficacy for patients.

To our excitement, our data showed that, 80% of the patients had a GRA score of 2 or higher among the 25 cases we collected at 6-month follow-up (Table 1). Compared to the similar treatment protocol in Jhang J. F. et al. (2023) which injected PRP intravascularly once a month for four times, we performed this once a month for three times. Our data (80% in 25 patients) revealed a significant increase in GRA ≥ 2 ratio than theirs (46.7% in 30 patients) at 6-month follow-up (p = 0.024). Besides, we also compared our VAS score (1.7 ± 1.6) to theirs (4.27 ± 3.11) and the result showed a significant improvement for the combination of PRP plus LI-ESWT at 1-month follow-up (p < 0.001), although their study showed no significant difference to ours at 6-month follow-up in VAS score. Moreover, the ICSI and ICPI all showed a significant decrease in our combined treatment at 1-month and 6-month follow-ups compared to their PRP-alone group, but the baseline showed no significant differences in these two groups at the values we investigated. Even though it may not entirely represent the PRP-alone group to our study, we believe it still holds significant reference value, as the treatment frequency in that group was even one session more.

Moreover, Jhang et al. demonstrated that LI-ESWT treatment for 8 weeks was simi-lar to our protocol (Jhang L. S. et al., 2023). They assessed ICSI, 3-day voiding diary, and urodynamic study. Our data at 1-month follow-up revealed no significant difference in 3-day voiding diary, although the MCC in our study showed significant increase whereas the results in their study did not show such increase. In the meantime, compared to LI-ESWT alone, PRP plus LI-ESWT showed better efficacy on the short-term effect of ICSI at the 1-month follow-up. Additionally, the therapeutic effect of PRP plus LI-ESWT in the short-term efficacy might have come from PRP’s role in promoting tissue repair or the an-ti-inflammatory effect of LI-ESWT. We also observed that only ICIQ showed a significant improvement at the 6-month follow-up, which might indicate the need for multiple treatment sessions. Furthermore, PRP + LI-ESWT showed superior short-term ICSI improvement compared to LI-ESWT monotherapy, suggesting that PRP could play a key role in reducing bladder inflammation and sensory hypersensitivity, although only ICIQ showed significant improvement at the 6-month follow-up, revealing the need for multiple treatment sessions to sustain long-term efficacy.

### 4.1 Comparison of PRP plus LI-EWST to PRP alone in LUTs

No study has discussed the effect of PRP on IC/BPS patients in urinary-related questionnaires, including OABSS, UDI-6, IIQ-7, and ICIQ, although some studies have investigated the effect of PRP on other lower urinary tract symptoms (LUTs) in urinary-related questionnaires (Jiang et al., 2022; Long et al., 2021; Saraluck et al., 2024; Athanasiou et al., 2021). The pilot study made by Athanasiou et al. (2021) showed that two injections of PRP for 6 weeks revealed significant improvement at the 3-month follow-up and a further improvement at the 6-month follow-up. Some indexes including ICIQ, GRA were significantly increased at 3- and 6-month follow-up assessments. Be-sides, a reduction of urine loss was also reported (Athanasiou et al., 2021).

Besides, our team also investigated the effects of PRP on SUI, finding that urinary-related questionnaires such as ICIQ-SF, UDI-6 and, IIQ-7 all showed significant change at 1- and 6-month follow-up assessments. The urodynamics studies also revealed that PRP treatment could significantly increase RU and FS in SUI patients (Long et al., 2021). Compared to our current study, it seems that PRP alone showed no difference from PRP plus LI-EWST; however, the baseline of IC/BPS patients is relatively higher in most indexes (POPDI-6, IIQ-7, UDI-6) and the pathological mechanisms of IC/BPS are complex. Saraluck et al. (2024) also combined PRP and pelvic floor muscle training (PFMT) and their data revealed that PRP plus PFMT showed better efficacy in symptoms of SUI at 2- and 5-month follow-up assessments (Saraluck et al., 2024). Our data showed that PRP plus LI-ESWT revealed a significant effect on UDI-6, IIQ-7 and POPDI-6 at 1-month follow-up and ICIQ at 6-month follow-up (Table 1). Except for the questionnaires, some studies also investigated the effects of PRP or LI-ESWT on urodynamics.

### 4.2 Effect of PRP or LI-ESWT alone on urodynamics studies in LUTs

Jiang et al. (2021) revealed that four injections within 3 months of PRP treatment could significantly increase abdominal leak point pressure (ALPP) and functional profile length (FPL) while showing no effect on other urodynamic parameters at 3-month follow-up (Jiang et al., 2021). They suggested that higher ALPP represents an increase in urethral resistance; meanwhile, longer FPL means increased external sphincter volume, while in fact, our data also showed that PRP plus LI-ESWT was significantly effective at 6-month follow-up (Table 3). The relationships between MCC and ALPP or FPL remain unclear; however, Kadano et al. (2014) investigated the urodynamic effects of urinary incontinence after robot-assisted radical prostatectomy using multivariate analysis to analyse urine loss ratio (ULR) versus urodynamics parameters including MCC, MUCP and, ALPP and determined that ULR was linearly correlate to these three indicators (Kadono et al., 2014).

Our study demonstrated significant short-term efficacy of PRP plus LI-ESWT in treating IC/BPS, with improvements observed in symptom scores, voiding parameters, and maximum cystometric capacity (MCC). The increase in MCC suggests improved bladder storage function and reduced hypersensitivity, supporting the hypothesis that PRP + LI-ESWT modulates sensory pathways involved in IC/BPS pathophysiology. MCC is particularly relevant as it directly reflects functional bladder capacity and aligns closely with patient-reported outcomes such as the GRA scale. Previous studies have shown that many pharmacologic therapies increase MCC, and our results are in line with these findings.

To provide context, Shen et al. (2023) reported that ESWT significantly increased total bladder capacity at the 3-month follow-up in patients with underactive bladder, but not at 1 month. In contrast, our study demonstrated an earlier MCC improvement after PRP + LI-ESWT, suggesting a potential synergistic effect and faster onset of benefit (Shen et al., 2023).

While our study demonstrates significant short-term efficacy of PRP plus LI-ESWT in treating IC/BPS, we acknowledge the inherent limitations of the single-arm design. The absence of a control group limits the ability to differentiate the effects of the combined therapy from potential placebo effects or natural disease progression while, the small sample size might reduce the statistical power and generalizability of our findings. To address these limitations, we compared our results with prior studies investigating PRP or LI-ESWT alone. This comparative analysis provides context and highlights the potential advantages of the combined therapy. While, such comparisons cannot fully re-place the rigor of a randomized controlled trial (RCT), future studies are required that include a control group, such as patients receiving PRP or LI-ESWT alone, or a placebo group, to validate the synergistic effects observed in our study. A larger sample size and extended follow-up periods would also help to establish the long-term efficacy and safe-ty of the combined therapy. We believe that our study provides valuable preliminary evidence for a novel approach to treating IC/BPS, which can inform the design of future RCTs.

The increase in maximum cystometric capacity (MCC) after PRP + LI-ESWT suggests an improvement in bladder storage function, which is a primary goal in IC/BPS management. Results of a previous study illustrated that almost all drugs could increase MCC after increasing the dosage followed by oxybutynin, solifenacin, tolterodine, trospium chloride and propiverine (Xu et al., 2022). MCC is more clinically relevant because it directly reflects functional bladder capacity which is an important determinant of patient-reported quality of life. Detrusor pressure and bladder compliance, while useful, might not capture symptom relief as effectively as MCC, which aligns with subjective patient-reported outcomes such as GRA scale. Improvements in MCC suggest reduced bladder hypersensitivity, supporting the hypothesis that PRP + LI-ESWT could modulate sensory path-ways involved in IC/BPS pathophysiology. Given these findings, MCC should be considered a key outcome measure in future IC/BPS trials assessing regenerative therapies.

IC/BPS is a complex chronic condition characterized by multifactorial pathophysiology. Urothelial dysfunction and disruption of the glycosaminoglycan (GAG) layer allow urinary solutes to penetrate the bladder wall, triggering inflammatory responses. This process is further amplified by mast cell activation and the release of pro-inflammatory cytokines, contributing to tissue damage and pain. Chronic inflammation may also lead to central sensitization and neuroinflammation, resulting in persistent pain and hypersensitivity.

**FIGURE 2:**
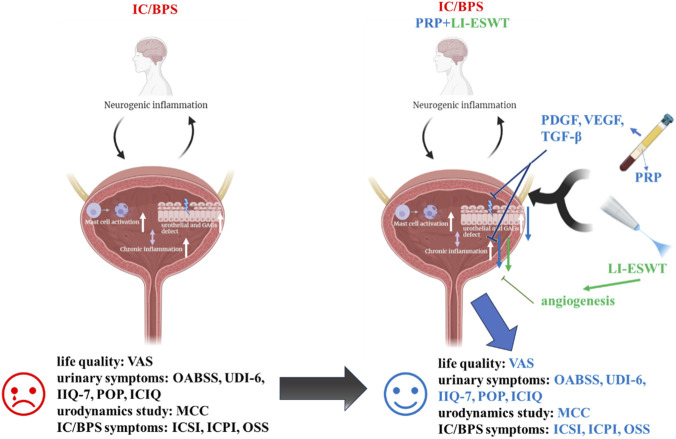
Schematic diagram of mechanism of action of platelet rich plasma (PRP) plus low-intensity extracorporeal shockwave therapy (LI-ESWT) on IC/BPS patients.

From a biological perspective, PRP promotes tissue repair and exerts anti-inflammatory effects through the release of growth factors, while LI-ESWT enhances local blood flow, angiogenesis, and tissue regeneration. The combination of these two modalities may therefore provide synergistic benefits by targeting both tissue healing and inflammatory pathways. In addition, LI-ESWT may facilitate tissue regeneration through mechanotransduction and enhance the bioavailability of PRP-derived growth factors, potentially contributing to their synergistic effects (Figure 2).

Although our study demonstrated significant symptomatic and functional improvement following PRP plus LI-ESWT treatment, the effects began to diminish by the 6-month follow-up in several domains. This reduction in therapeutic benefit may be at-tributed to several biological factors. First, the bioactive components in PRP, including growth factors have a limited half-life and may not sustain long-term tissue remodelling unless repeatedly administered. Second, the anti-inflammatory effects induced by LI-ESWT and PRP may be temporary, especially in the context of a chronic relapsing condition like IC/BPS, which is characterized by persistent urothelial dysfunction, mast cell activation, and neurogenic inflammation. Finally, the absence of ongoing stimulus may lead to a gradual regression of angiogenesis and tissue repair. Similar patterns have been observed in musculoskeletal and pelvic pain disorders treated with regenerative medicine, where maintenance therapy has been shown to prolong symptom control (Prost et al., 2024; Galasso et al., 2020). These considerations highlight the potential need for periodic PRP or LI-ESWT sessions to sustain therapeutic effects over time, and future studies should investigate optimal treatment intervals for long-term efficacy.

Our study demonstrated significant short-term efficacy of PRP plus LI-ESWT in treating IC/BPS patients, with improvements observed in multiple clinical parameters at 1 month. However, a decline in therapeutic benefits was noted by 6 months, raising the question of whether maintenance sessions are needed. While there is currently no standardized protocol for long-term management using PRP or LI-ESWT in IC/BPS, in-sights can be drawn from established guidelines and clinical practices in chronic urological conditions. Homma et al. (2020) highlighted the importance of periodic interventions, particularly in cases where symptoms fluctuate or relapse occurs over time (Homma et al., 2020). The guidelines mentioned intravesical therapy, hydrodistension and neuromodulation as treatments that might require repeated sessions to sustain efficacy. Similarly, in LI-ESWT treatment for chronic prostatitis/chronic pelvic pain syndrome, it is suggested that ex-tending treatment duration beyond the initial protocol could enhance symptom relief and sustain therapeutic benefits (Sokmen and Comez, 2023). In musculoskeletal conditions, repeated LI-ESWT sessions are commonly used to prevent symptom recurrence and maximize long-term outcomes, and given the chronic and relapsing nature of IC/BPS, a similar maintenance regimen involving periodic PRP or LI-ESWT sessions could well help sustain symptom relief.

While our study demonstrated significant short-term benefits, the gradual reduction in therapeutic effects by 6 months underscores the need for further research. It is rea-sonable to consider repeating the PRP + LI-ESWT protocol biannually to sustain clinical benefits, especially in patients with symptom relapse. While our study did not stratify outcomes by age or disease duration, it is possible that younger patients or those with a shorter history of IC/BPS may exhibit greater responsiveness due to higher tissue regenerative potential. Besides, the follow-up period of the current literature on PRP combined with LI-ESWT is up to 6 months. The average age of patients in the research are mostly around 50 years old. Hence, further studies are needed to validate subgroup-specific responses and determine individualized maintenance schedules. We propose a future randomized controlled trial (RCT) with four parallel arms: (1) PRP alone, (2) LI-ESWT alone, (3) combination therapy, and (4) placebo control. All patients would receive treatments over a similar duration and undergo uniform outcome assessments at baseline, 1, 6, and 12 months. A follow-up period exceeding 12 months would be essential to evaluate the sustainability of symptom improvement and treatment durability. Such a design would allow for direct comparison of monotherapies versus combination therapy, as well as estimation of placebo effects, thereby clarifying the true therapeutic contribution of each modality. There is the issue of cost, the cost of a full PRP course is approximately 1,000–2,000 USD, while a complete LI-ESWT course is about 1,500–2,500 USD in general. Com-bination therapy may thus cost 2,500–4,500 USD per treatment cycle. However, if combi-nation therapy achieves better short-term efficacy and reduces the need for repeated therapies compared to monotherapy, it could offer potential cost-effectiveness by lowering cumulative healthcare utilization over time. Formal health economic analyses are warranted.

This study has several limitations. First, the absence of a control group limits the ability to differentiate treatment effects from potential placebo effects and precludes definitive causal inference. Second, the relatively small sample size (n = 25) may reduce statistical power and limit generalizability; however, this study was designed as an exploratory pilot investigation to provide preliminary clinical insights. Third, the pre–post design without randomization may introduce selection bias. In addition, the absence of blinding may increase the risk of placebo effects and observer bias, particularly in patient-reported outcomes. Fourth, the follow-up duration of 6 months may be insufficient to capture the long-term durability of treatment effects in a chronic condition such as IC/BPS. Fifth, the LOCF approach may be associated with false positive results and may exaggerate the treatment effects. Finally, the inclusion of only female patients from a single center may limit the generalizability of our findings to broader populations, including male patients and different clinical settings. Therefore, the findings of this study should be interpreted as preliminary and hypothesis-generating. Future studies are warranted to include randomized controlled designs, larger sample sizes, and longer follow-up periods to validate these results and to determine the optimal treatment protocols, including the frequency and maintenance strategies of PRP and LI-ESWT.

## 5 Conclusion

In this exploratory study, the combination of platelet-rich plasma (PRP) and low-intensity extracorporeal shock wave therapy (LI-ESWT) demonstrated promising short-term efficacy in patients with IC/BPS. The treatment was associated with improvements in symptom burden, quality of life, and functional bladder capacity, supporting the potential benefit of this combined regenerative approach.

However, the durability of these effects appeared limited, suggesting that repeated or maintenance treatment strategies may be required to sustain clinical outcomes. These findings highlight the potential role of PRP plus LI-ESWT as a minimally invasive treatment option for IC/BPS, particularly in patients with refractory symptoms.

Further well-designed randomized controlled trials with larger sample sizes and longer follow-up are needed to confirm these findings, optimize treatment protocols, and better define the long-term clinical value of this combination therapy.

## Data Availability

The original contributions presented in the study are included in the article/supplementary material, further inquiries can be directed to the corresponding author.
